# Supplementary material for: Influence of atomic site-specific strain on catalytic activity of supported nanoparticles
Source: Nat Commun. 2018 Jul 13;9:2722. doi: 10.1038/s41467-018-05055-1 (PMC6045581; doi:10.1038/s41467-018-05055-1)
Supplement: Supplementary file 1 — Supplementary Information [file 41467_2018_5055_MOESM1_ESM.pdf]

Supplementary Information

Influence of Atomic Site-Specific Strain on Catalytic Activity of Supported Nanoparticles

Nilsson Pingel et al.

Supplementary Note 1: Additional STEM data

The raw and non-rigidly registered image series for NP 1 are shown in Supplementary Movies 1 and 2 (8x sped up). Supplementary Figures 1a-c show a precision analysis within the reference area of the largest grain as a function of the number of averaged images. This shows that image precision does not significantly improve after 50-60 images, and that the precision behaviour closely follows the $1/\sqrt{n}$ prediction, where n is the noise level. Supplementary Figure 1d shows NP 1 in a different colour scale to reveal the location of the support material and to highlight the NP-support interface. Supplementary Figure 2 labels the electron beam direction and the crystallographic planes in each grain of the investigated NPs. Supplementary Figure 3 shows a top-view high-precision image and corresponding displacement and strain maps of a Pt NP on alumina, for comparison to the side-view images shown in the main text. This top-view data reveals no asymmetric strain behaviour at the NP surface, in contrast to the asymmetric strain observed in the side-view data. Strain histograms of NP 2 – NP 4 are presented in Supplementary Figure 4.

Supplementary Note 2: Bond angle maps

In addition to projected strain maps, projected bond angle maps are also useful for quantifying and visualising local NP lattice distortions. These are measured by calculating the deviation of nearest-neighbour bond angles from the average bond angle in the reference area of each grain, in each crystallographic direction separately. These reveal continuous lattice plane bending patterns across the grains in each NP and strong localised bending at specific surfaces and interfacial sites (Supplementary Figure 6). In particular, the projected bond angle maps from the decahedral NP show a continuous bending of the lattice planes around the decahedral NP (Supplementary Figure 6l), which accommodates the angular gap created from assembling the tetrahedral subunits. The observed bending angles of 0.9 – 1.7° per grain, adding up to a total of 7.1°, are consistent with previous measurements and theory[1,2].

Supplementary Note 3: Activity of strain patterns

The activity of each strain pattern in Figure 4 can be understood by analysing how strain affects facets, edges and corners, respectively. Supplementary Table 2 shows the average strain and TOFs normalised to the TOF for the unstrained NP (Pattern 0). In general, the highest activities are obtained when {111} and {100} facets are expanded, and the corners and edges are slightly compressed. Pattern 3 shows that straining only edges and corners lowers the TOF on the facets, which emphasizes that kinetic coupling is crucial to understand the catalytic activity of nanoparticles. Note that the relative activity is temperature dependent, as the chemical potential of CO and O_2 varies with temperature.

Supplementary Note 4: Rate constants

For adsorption reactions, the rate-constants are evaluated with the following expression:

$$W_{\mathrm{i}j}^{\mathrm{ads}} = \frac{s_{\mathrm{i},j}p_{\mathrm{i}}}{\sqrt{2\pi M_i k_{\mathrm{B}}T}} \tag{1}$$

Where i is the species, j is a site-index, p_{i} is the pressure, $s_{\mathrm{i},j}$ is the sticking coefficient, and M_i is the mass of the impinging molecule. Relevant sticking coefficients have been reported[3,4], and we use s_{O_2} = 0.1 on the facets and s_{O_2} = 1 on corners and edges. Similarly, the CO sticking coefficient was set to s_{CO} = 0.9 on the facets and s_{CO} = 1 on corners and edges. The sticking coefficients describe eventual molecular precursor states.

The rate constants of desorption were calculated using the adsorption rate constants and the equilibrium constants:

$$W_{\mathrm{i},j,\alpha}^{\mathrm{des}} = \frac{W_{\mathrm{i},j,\alpha}^{\mathrm{ads}}}{K_{\mathrm{i},j,\alpha}}, \quad K_{\mathrm{i},j,\alpha} = \exp[\frac{-E_{\mathrm{i},j,\alpha}^{\mathrm{ads}} - T\left(S_{\mathrm{i}}^{\mathrm{gas}} - S_{\mathrm{i},j}^{\mathrm{ads}}\right)}{k_{\mathrm{B}}T}] \tag{2}$$

Here, i is the species, j is the site, and α is the state of the system. Furthermore, $E_{\mathrm{i},j,\alpha}^{\mathrm{ads}}$ is the adsorption energy, $S_{\mathrm{i}}^{\mathrm{gas}}$ is the entropy of the gas-phase molecule, $S_{\mathrm{i},j}^{\mathrm{ads}}$ is the entropy of the adsorbate state, and $K_{\mathrm{i},j,\alpha}$ is the equilibrium constant. $S_{\mathrm{i}}^{\mathrm{gas}}$ is evaluated using the ideal gas approximation[5], and $S_{\mathrm{i},j}^{\mathrm{ads}}$ is evaluated in the Harmonic Approximation[5]. $E_{\mathrm{i},j,\alpha}^{\mathrm{ads}}$ scales with the generalised coordination number[6,7], and a scaling relation is fitted to results on extended surfaces (see Supplementary Note 5).

The rate constant for CO_2 formation ($CO* + O* \rightarrow CO_2(g)$) is found by Transition State Theory (TST)[8].

$$W_{j,\alpha}^{r} = \frac{k_{\mathrm{B}}T}{h}\frac{Z^{TS}}{Z^{IS}}\exp\left(\frac{-E_{j,\alpha}}{k_{\mathrm{B}}T}\right), \tag{3}$$

where $E_{j,\alpha}$ is the activation energy of CO oxidation, Z^{TS} is the partition function in the transition state, and Z^{IS} is the partition function in the initial state. The partition functions are evaluated on the (111) surface in the Harmonic Approximation[5] using calculated vibrational frequencies. $E_{j,\alpha}$ is evaluated by a calculated BEP relation between the transition state and co-adsorbed CO and O[9].

Diffusion is treated similarly with TST as described earlier[5]. The diffusion barriers of CO and O are calculated on Pt(111) in a (3x3) cell. A diffusion barrier of 0.08 eV is obtained for CO diffusing from the bridge to the fcc site, and a barrier of 0.58 eV is found for O moving from the fcc to the hcp site. In the Monte Carlo simulations, diffusion barriers between different types of sites are adjusted by the differences in adsorption energies. KMC simulations with very different rates are not computationally feasible, hence we increase the diffusion barriers of CO while ensuring convergence (see Supplementary Note 6). This approach has been discussed and applied previously[10-12].

Supplementary Note 5: Reaction energy landscape

The reaction energy landscape is based on scaling relations, with the Pt(111) surface as a point of origin from where energies are scaled in the simulations. The generalised coordination number ($\overline{\text{CN}}$) describes the coordination of the site and it includes contributions from the neighbouring sites. In this manner an inner facet has a different $\overline{\text{CN}}$ than a facet-site next to an edge. This is illustrated in Supplementary Figure 8a, where $\overline{\text{CN}}$ is plotted for a 5.2 nm NP. The generalized coordination number is a descriptor for adsorption energies on nanoparticles and extended surface[6,7]. We used the model surfaces presented in Supplementary Figures 8b-c to find the linear relation between $\overline{\text{CN}}$ and the adsorption energies. The result of this is presented in Supplementary Figure 9a.

The d-band centres for a transition metal system are another descriptor for the adsorption energies[13]. Straining a system modifies the d-band and consequently gives rise to a scaling relation. In Supplementary Figure 9b the scaling relation between adsorption energies and strain is shown. The strain is only compressive as expansive strain modifies the applied slab model. In the Monte Carlo simulations, the compressive strain was used to extrapolate linearly into the expansive regime.

The Transition State (TS) for CO_2 formation ($CO + O \rightarrow CO_2$) scales with the adsorption energies of CO and O. We calculated the Brøndsted-Evans-Polanyi relation for the present case using strained surfaces, see Supplementary Figure 9c. The slope of this BEP relation is used to evaluate the reaction energies during the Monte Carlo simulations.

We include repulsive interactions between nearest-neighbour adsorbates. The repulsions are calculated as the difference in average adsorption energy between a (2x2) surface cell with one and two adsorbates. The CO-CO repulsions were determined to be 0.19 eV per neighbour, which was found as the difference between CO(hcp)+CO(fcc) and CO(fcc). The O-O repulsions are 0.32 eV per neighbour and are determined as the difference between O(fcc)+O(fcc) and O(fcc). The CO-O interactions are 0.3 eV and are determined as the difference between CO(fcc)+O(fcc) and the adsorbate in separate cells with CO(fcc) and O(fcc). To account for the geometry of the edges and corners, the repulsions were multiplied by 0.5 on these sites. Supplementary Table 3 summarizes the simulation parameters.

Supplementary Note 6: Simulation convergence

The diffusion barrier of CO is much lower than the other barriers of the simulation. Thus, for technical reasons it is useful to raise this barrier while ensuring convergence in the kinetics. Such a test is reported in Supplementary Figure 10a where the turnover frequency is plotted against the CO diffusion barrier. Based on these results, we chose to perform the simulation with a barrier of 0.58 eV which ensures convergence. The simulations are run until a steady-state is reached, which is determined by when the coverages reach equilibrium. In Supplementary Figure 10b, the CO coverage is reported for a simulation. The simulations were initiated from a fully CO-covered NP.

Supplementary Note 7: Turnover frequency of sites

Supplementary Figure 11 shows the various sites TOF versus average strain of the given sites. The TOF is normalized to the TOF of the unstrained NP. The plot suggests that there is an optimal strain for each site. The plot suggests that at the present conditions, the facets benefit from expansive strain, whereas the edges and corners benefit from compression. The relation resembles a Sabatier volcano plot. However, as there are many sites and the kinetics between these sites is coupled, the picture is slightly more complex.

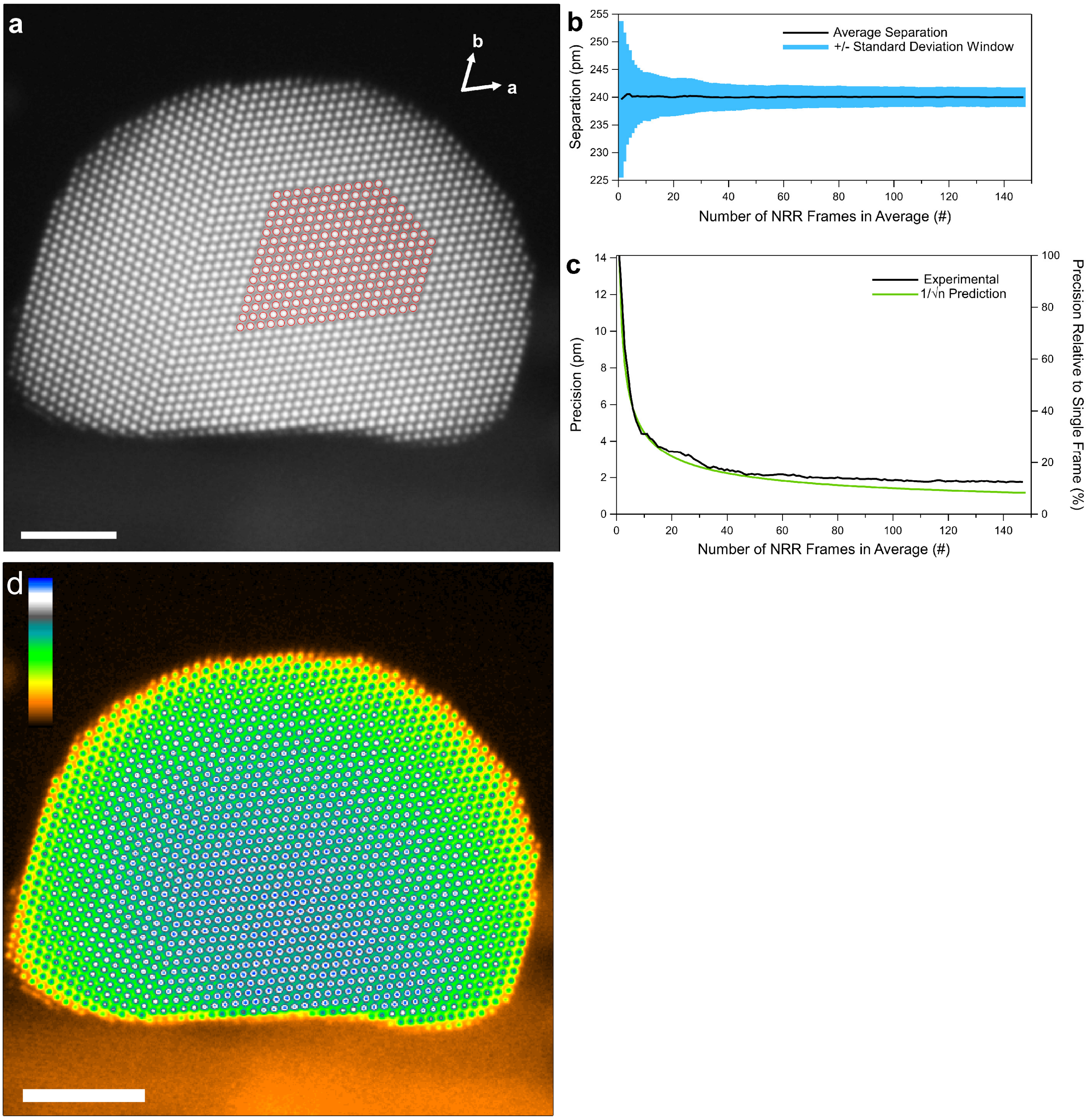

Supplementary Figure 1: Precision enhancement resulting from NRR and averaging. (a) Non-rigidly registered and averaged HAADF STEM image of the same Pt NP and data shown in Figure 1a. The atomic columns used in this precision analysis are marked by red open circles. All the interatomic separations within the two {111} planes marked by the a and b arrows are used. (b) The black curve indicates the average separation distance between the atomic columns within the two {111} planes as a function of the number of frames used in the averaged image after NRR. The blue window indicates the +/- standard deviation of these interatomic separations as a function of number of images used in the average. (c) The black line indicates the actual experimental precision (left axis) and the precision relative to the precision in the first NRR image (right axis) as a function of the number of NRR and averaged frames. The image precision is defined as the standard deviation in the measured interatomic separations. The green curve indicates the predicted $1/\sqrt{n}$ noise trend relative to a single frame (right axis) as a function of the number of frames in the average. (d) STEM image of NP 1 in a different colour scale. The lower part of the NP is in contact with the Al_2O_3 support while the upper part terminates to vacuum. The colour scale was chosen to increase the contrast of the Al_2O_3 support material. Each scale bar corresponds to 3 nm.

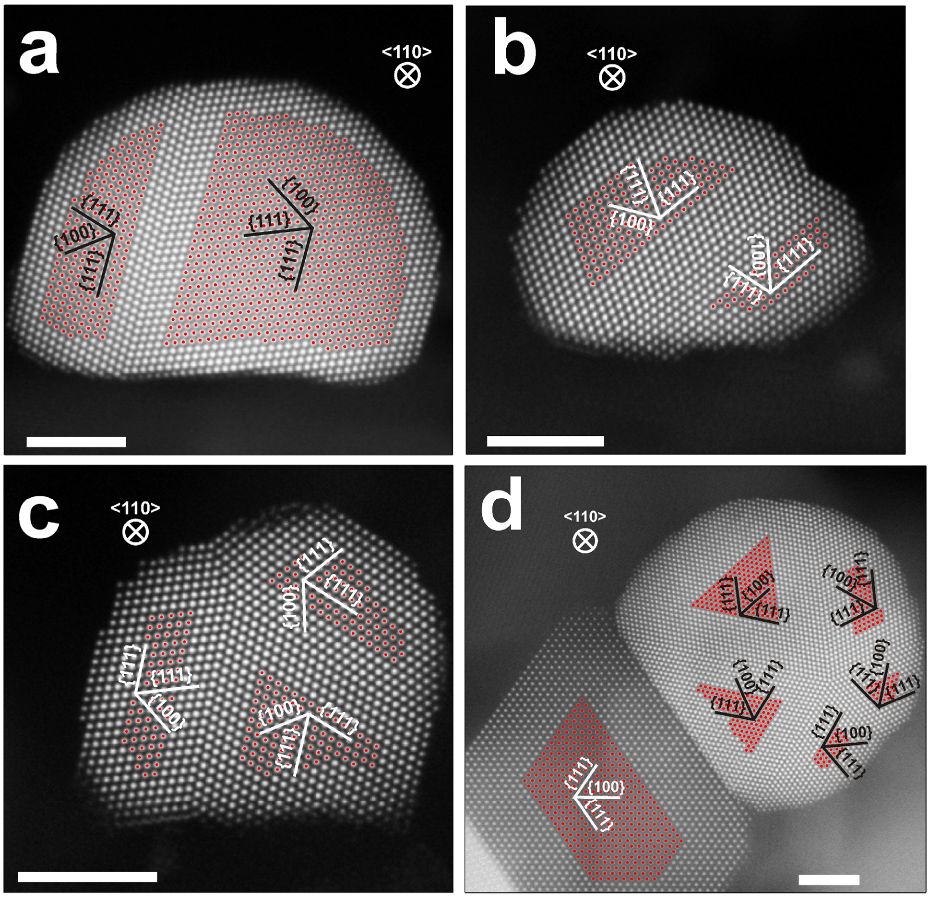

Supplementary Figure 2: Crystallographic planes and precision areas. For each dataset presented in Figure 1, the crystallographic planes that were used for strain measurements are marked in each grain of the Pt NPs. In each grain, the precision areas are marked by red dots on top of the atomic columns. The <110> electron beam direction is indicated. Each scale bar corresponds to 3 nm.

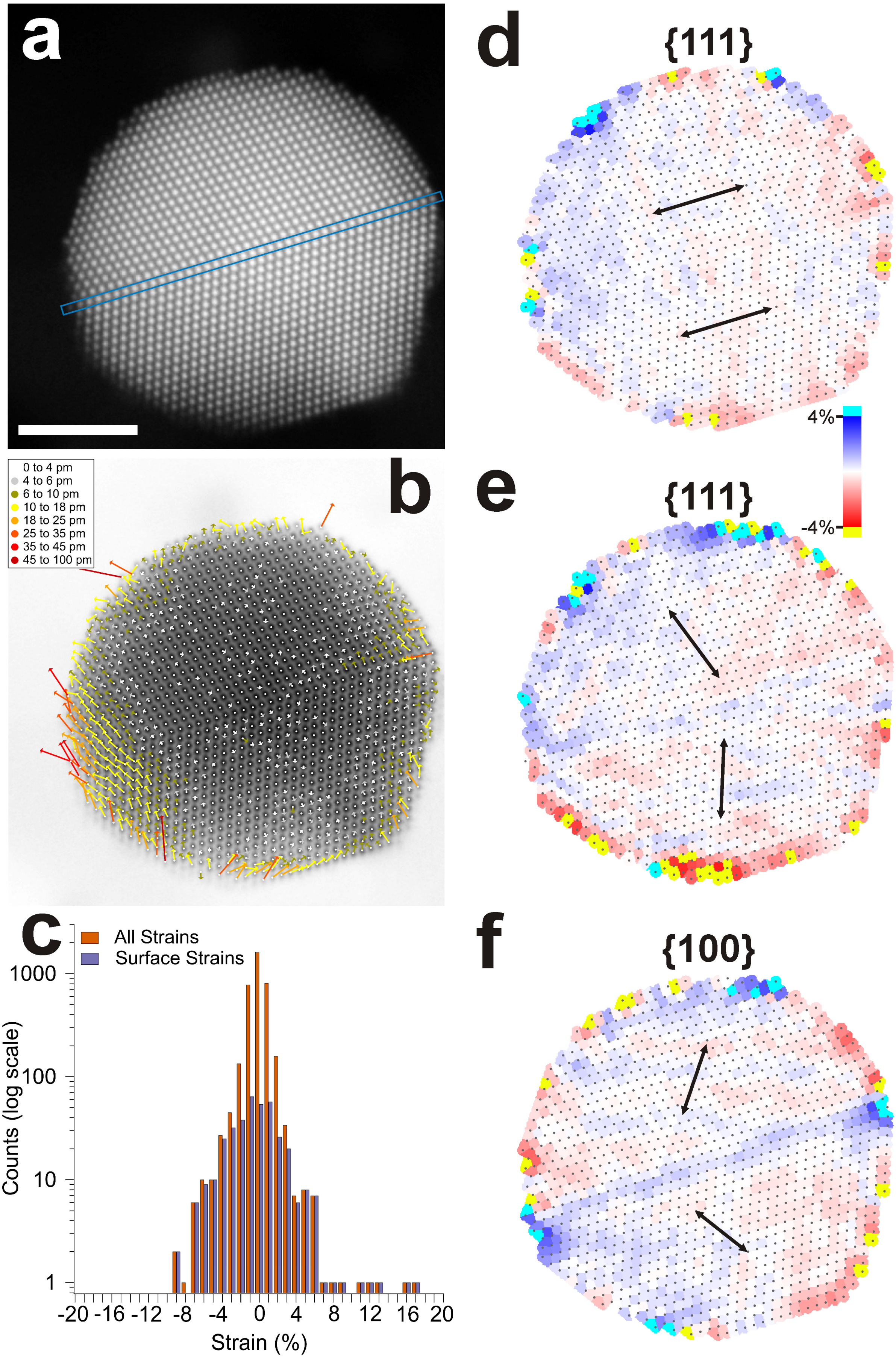

Supplementary Figure 3: Pt NP on alumina in top view. (a) Top-view high-precision STEM image, (b) displacement map, (d-f) strain maps and (c) strain histograms of a Pt NP on alumina for comparison with the side-view STEM images shown in Figure 1. The scale bar corresponds to 3 nm.

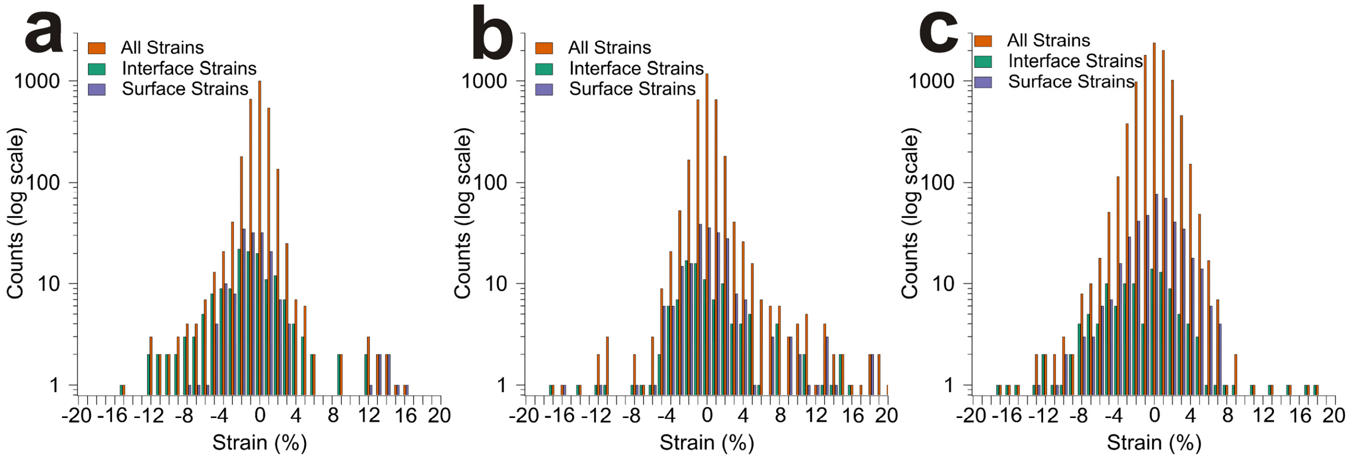

Supplementary Figure 4: Strain histograms. (a) NP 2, (b) NP 3, (c) NP 4.

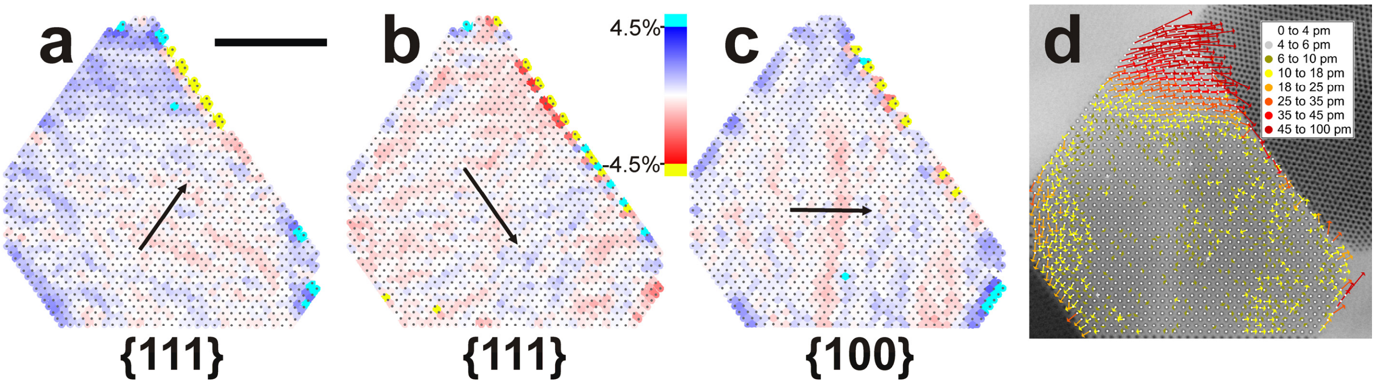

Supplementary Figure 5: Strain in ceria support. Strain maps (a-c) and displacement map (d) of the ceria grain shown in Figure 1g. Some atomic columns at the interface could not be uniquely assigned to either material with certainty, and might even be intermixed, so they are included here and in the maps in the main text. The scale bar corresponds to 5 nm.

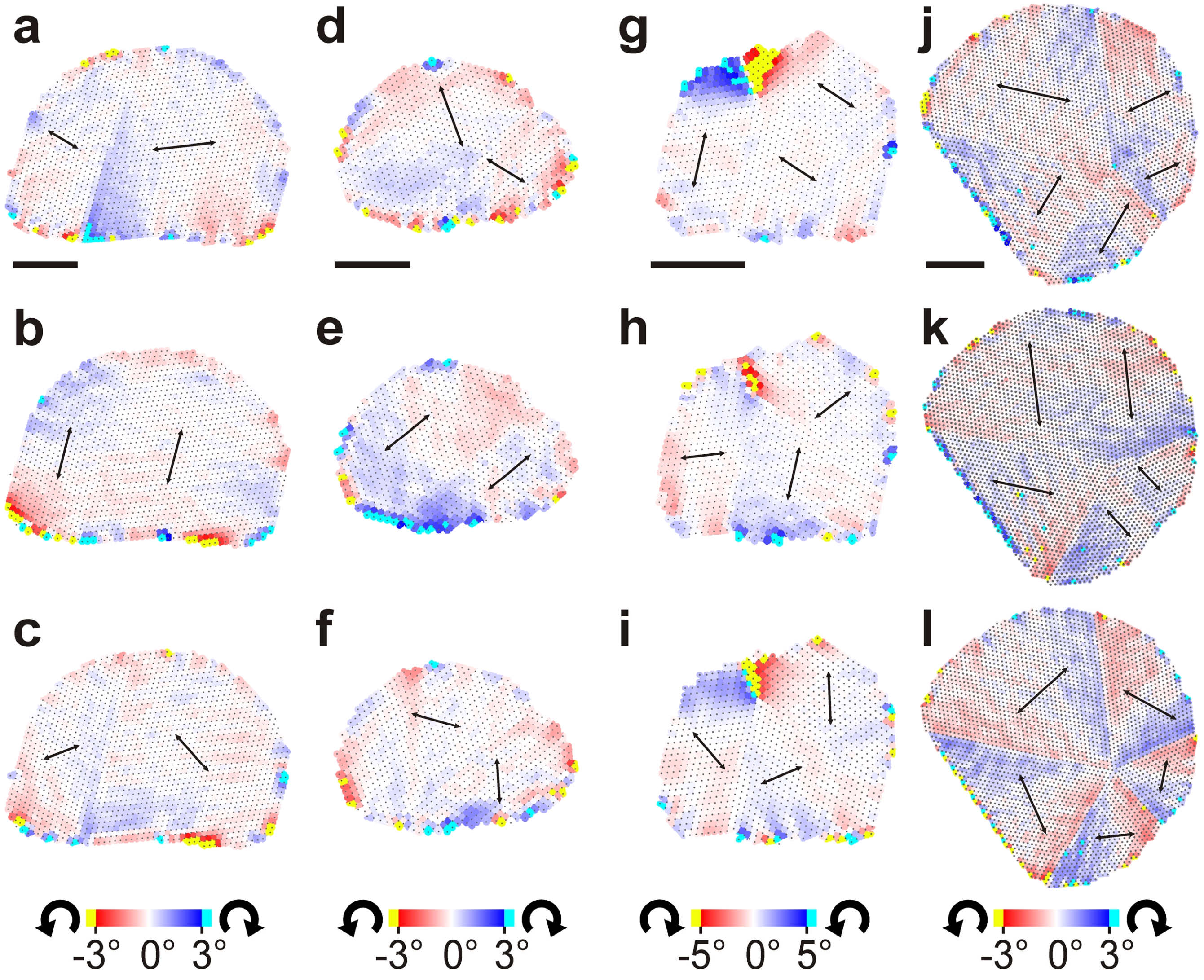

Supplementary Figure 6: Projected bond angle maps showing the deviation of nearest-neighbour bond angles from the average bond angle in the reference area of each grain. The arrows indicate the crystallographic planes in which the bond angles were measured. Note that rotation directions are inverted in subfigures g-i. (l) shows continuous bending of the lattice planes around the decahedral NP. The accumulated total bending angle of ~7.1° compensates for the angular gap that arises when assembling tetrahedral subunits. Each scale bar corresponds to 3 nm.

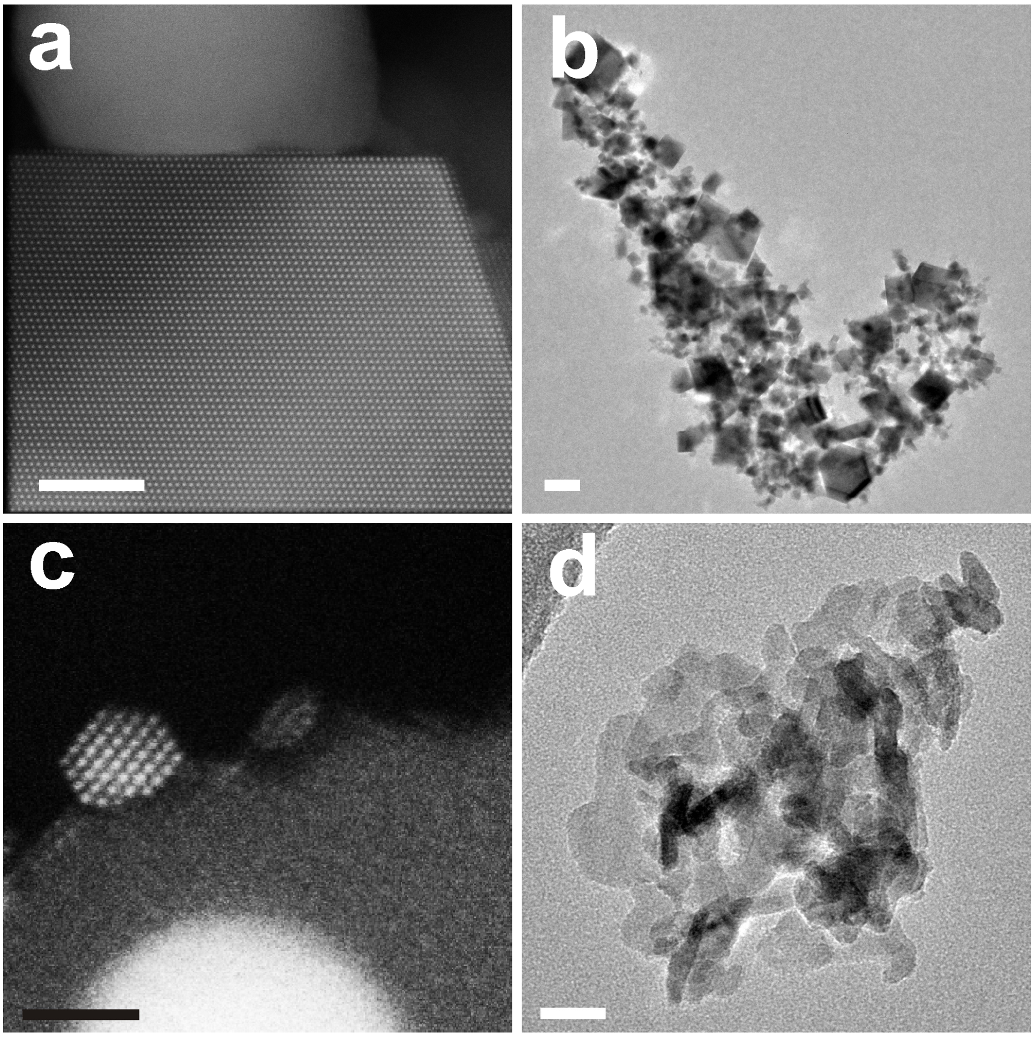

Supplementary Figure 7: Differences between the two support materials. **(**a) HAADF STEM image of a single-crystalline ceria grain with atomically flat facets. A Pt NP can be seen supported on the ceria grain. The scale bar corresponds to 5 nm. (b) TEM image of ceria nanopowder on a carbon film. Several large, faceted grains with extended flat facets can be seen. The scale bar corresponds to 50 nm. (c) HAADF STEM image of the edge of an alumina support particle with a few Pt NPs on it. The alumina edge appears rugged and corrugated. The scale bar corresponds to 2 nm. (d) TEM image of alumina nanopowder. The individual alumina grains appear rounded with mostly irregular shapes and no sharp surface facets. The scale bar corresponds to 20 nm.

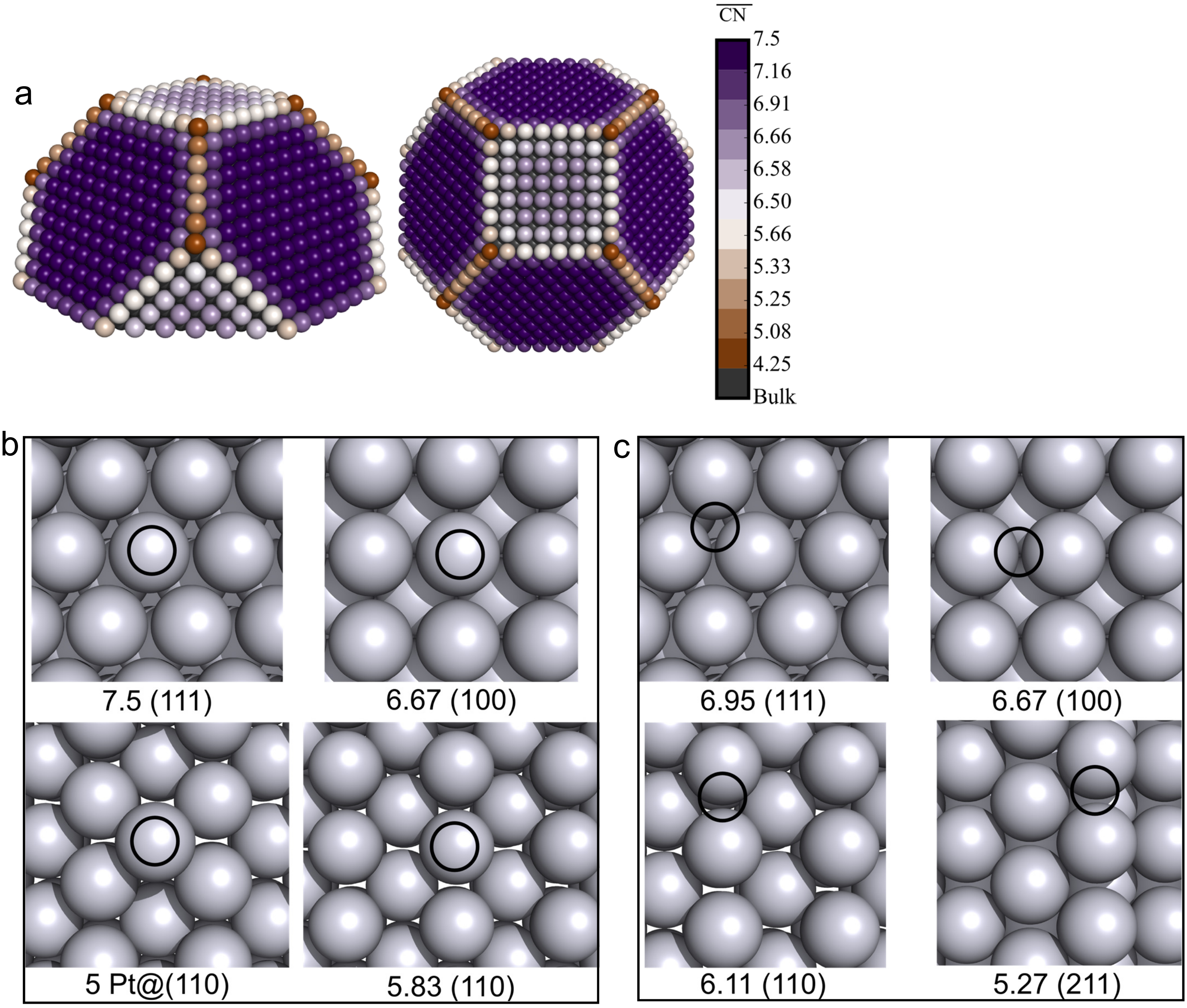

Supplementary Figure 8: Use of generalised coordination numbers. (a) The generalised coordination number for on-top sites of the truncated octahedron nanoparticle of 5.2 nm in diameter. (b-c) Model systems and generalised coordination numbers used to establish the scaling relations for (b) CO and (c) O.

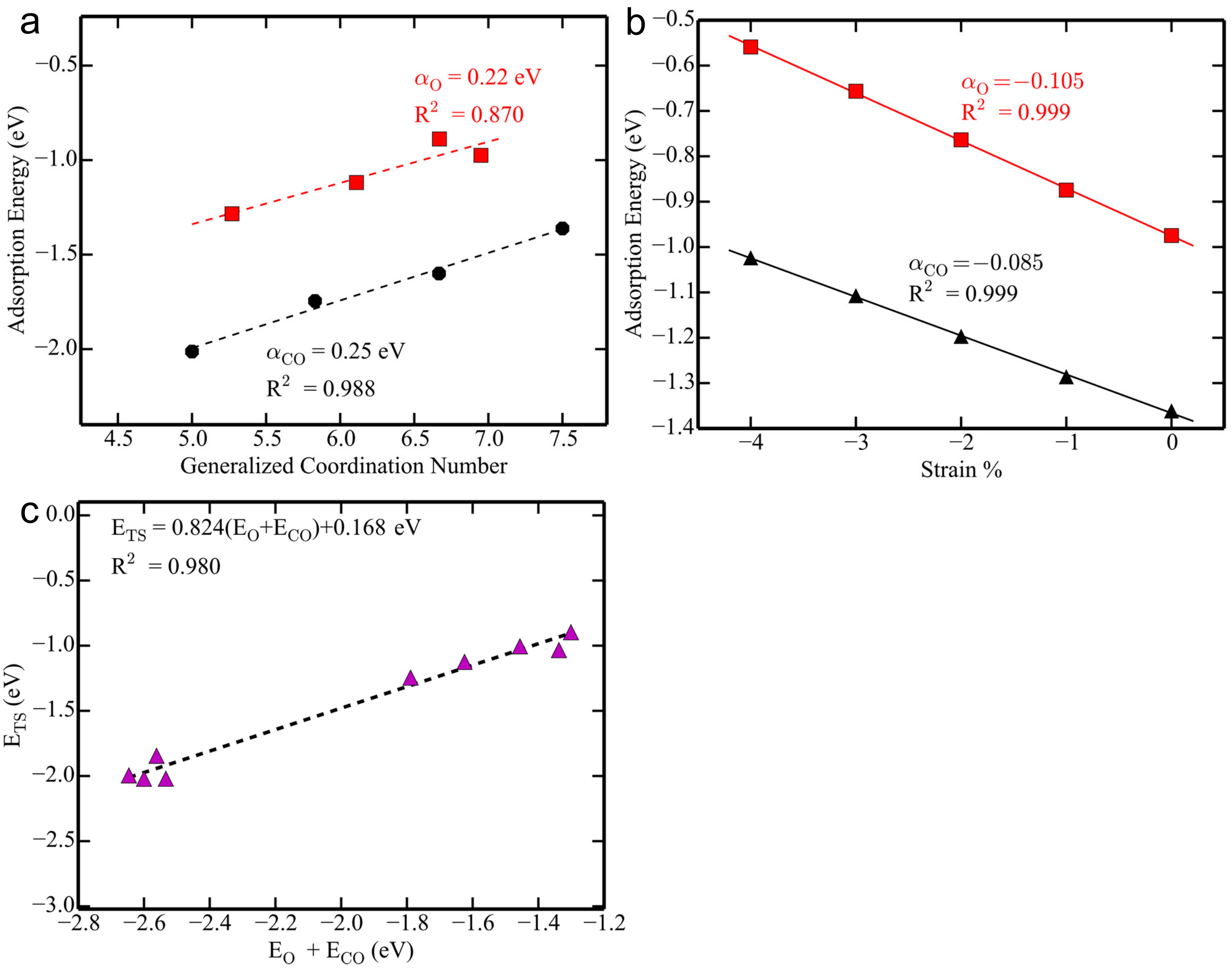

Supplementary Figure 9: Scaling relations. (a) The scaling relation of adsorption energies in the generalised coordination number. (b) Adsorption energy scaling relations on compressive strain, evaluated on Pt(111). (c) The Brøndsted-Evans-Polanyi (BEP) relation between the transition state energy and the adsorption energy of co-adsorbed CO and O.

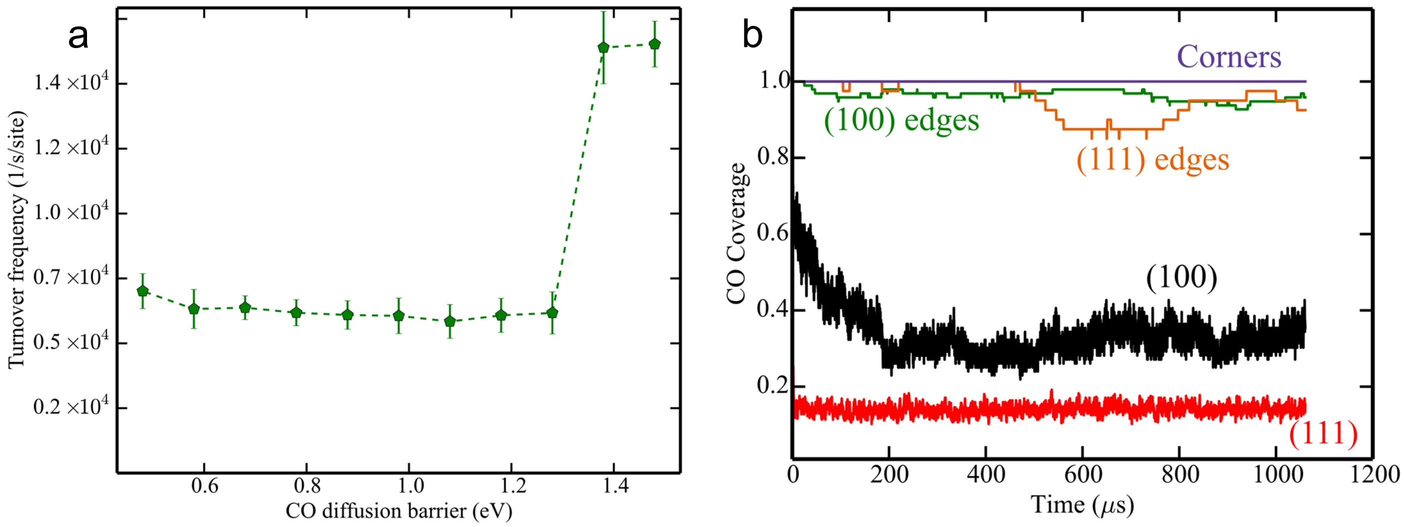

Supplementary Figure 10: Convergence of the simulations. (a) Convergence test with respect to the CO diffusion barrier. The calculated barrier is 0.08 eV. The error bars correspond to one standard deviation between 16 identically prepared simulations. (b) Convergence of the CO coverages in time. The CO pressure was 20 mbar, the O_2 pressure was 10 mbar, and the temperature was 600 K.

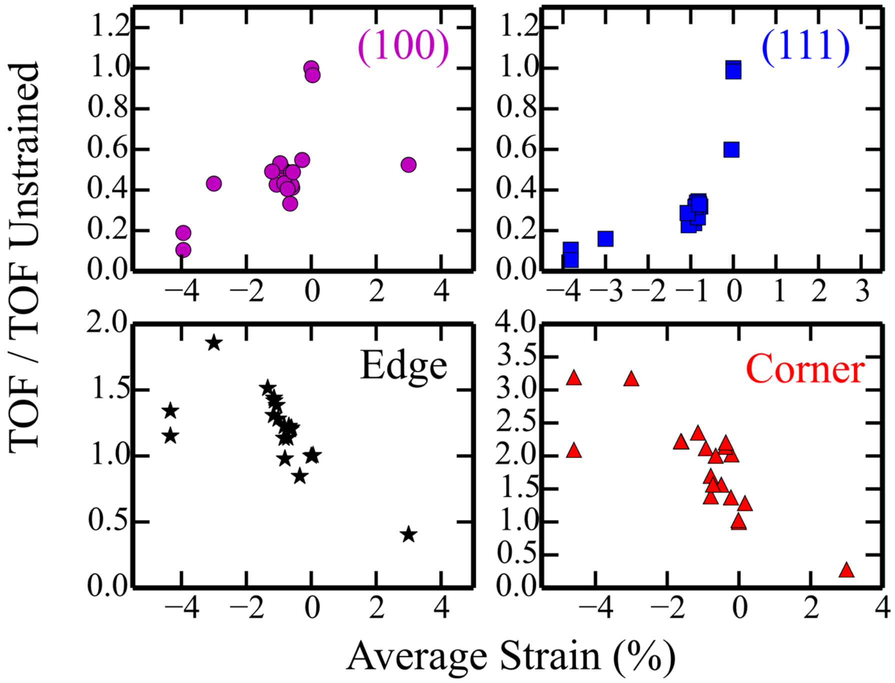

Supplementary Figure 11: Simulated TOF normalized to the TOF for the unstrained NP is shown as a function of average strain for the sites. Temperature 900 K, CO pressure 20 mbar, and O_2 pressure 10 mbar.

Figure	Image size	Number of frames	Pixel dwell time	Acquisition time	Average dose rate / $e^-/(A^2*s)$	Total dose / $e^-/(A^2)$	Acceleration voltage
1a	512x512 px	148	2 µs	94 s	610	$5.8x10^4$	200 kV
1c	512x512 px	200	2 µs	126 s	1050	$1.3x10^5$	300 kV
1e	512x512 px	103	3 µs	97 s	1050	$1.0x10^5$	300 kV
1g	1024x1024 px	78	2 µs	149 s	360	$5.3x10^4$	300 kV

Supplementary Table 1: Imaging parameters of all STEM experiments presented in this work. The electron probe current was ~3 pA for all measurements. The average dose rates were calculated by dividing the total doses by the acquisition times of the image series.

Pattern	1	2	3	4	5	6	7	8
δ{100}	3.00	-3.00	0.00	-0.52	-0.10	-0.42	-3.94	0.04
A{100}	1.67	0.26	0.74	0.27	0.49	0.36	0.06	0.94
δ{111}	3.00	-3.00	0.00	-0.86	-0.07	-0.79	3.82	0.00
A{111}	3.12	0.21	0.71	0.25	0.49	0.40	0.06	1.05
δ Edge	3.00	-3.00	-2.00	-0.99	-0.43	-0.56	-4.34	0.06
A Edge	0.53	0.62	1.39	0.47	0.63	0.60	0.23	0.98
δ Corner	3.00	-3.00	-2.00	-1.6	-1.33	-0.28	-4.60	-0.01
A Corner	0.31	1.01	1.5	0.84	0.91	0.79	0.62	1.02

Supplementary Table 2: The average strain in % (δ) for the patterns shown in Figure 4, and the corresponding TOFs divided by the TOF of the unstrained particle (A).

Parameter	Value
Reaction barrier	2.95 – 0.824 $(E_{\mathrm{O}} + E_{\mathrm{CO}})$
CO adsorption energy	−1.36 + 0.252 $(\overline{CN} - 7.5)$
O adsorption energy	−0.95 + 0.218 $(\overline{CN} - 7.5)$
Repulsion CO-CO	0.19
Repulsion O-O	0.32
Repulsion CO-O	0.30
Vibrational energies CO	10^{-3} x (17.8, 18.3, 36.3, 36.3, 39.7, 216.8)
Vibrational energies O	10^{-3} x (47.3, 47.6, 55.1)
Vibrational energies TS	10^{-3} x (7.3, 19.9, 36.4, 40.4, 50.5, 54.6, 65.1, 245)
Diffusion barrier CO	0.08
Diffusion barrier O	0.58
CO diffusion barrier addition	0.50
Sticking probability CO	facets: 0.9, edges and corners 1.0
Sticking probability O_2	facets: 0.1, edges and corners 1.0

Supplementary Table 3: Parameters used in the Kinetic Monte Carlo simulations. The energies are given in eV.

Supplementary References

1 Ino, S. Stability of multiply-twinned particles. *J. Phys. Soc. Jpn.* **27**, 941-953 (1969).

2 Johnson, C. L. *et al.* Effects of elastic anisotropy on strain distributions in decahedral gold nanoparticles. *Nat. Mater.* **7**, 120 (2007).

3 Campbell, C. T., Ertl, G., Kuipers, H. & Segner, J. A molecular beam investigation of the interactions of CO with a Pt(111) surface. *Surf. Sci.* **107**, 207-219 (1981).

4 Yeo, Y. Y., Vattuone, L. & King, D. A. Calorimetric heats for CO and oxygen adsorption and for the catalytic CO oxidation reaction on Pt{111}. *J. Chem. Phys.* **106**, 392-401 (1997).

5 Jørgensen, M. & Grönbeck, H. Adsorbate entropies with complete potential energy sampling in microkinetic modeling. *J. Phys. Chem. C* **121**, 7199-7207 (2017).

6 Calle-Vallejo, F., Martínez, J. I., García-Lastra, J. M., Sautet, P. & Loffreda, D. Fast prediction of adsorption properties for platinum nanocatalysts with generalized coordination numbers. *Angew. Chem. Int. Ed.* **53**, 8316-8319 (2014).

7 Calle-Vallejo, F., Loffreda, D., KoperMarc, T. M. & Sautet, P. Introducing structural sensitivity into adsorption–energy scaling relations by means of coordination numbers. *Nat. Chem.* **7**, 403-410 (2015).

8 Eyring, H. The activated complex and the absolute rate of chemical reactions. *Chem. Rev.* **17**, 65-77 (1935).

9 Falsig, H. *et al.* Trends in the catalytic CO oxidation activity of nanoparticles. *Angew. Chem. Int. Ed.* **47**, 4835-4839 (2008).

10 Jansen, A. P. J. *An introduction to kinetic monte carlo simulations of surface reactions*. 35-6, 211-213 (Springer Berlin Heidelberg, 2012).

11 Yang, L., Karim, A. & Muckerman, J. T. Density functional kinetic monte carlo simulation of water–gas shift reaction on Cu/ZnO. *J. Phys. Chem. C* **117**, 3414-3425 (2013).

12 Piccinin, S. & Stamatakis, M. CO oxidation on Pd(111): a first-principles-based kinetic monte carlo study. *ACS Catal.* **4**, 2143-2152 (2014).

13 Hammer, B. & Norskov, J. K. Why gold is the noblest of all the metals. *Nature* **376**, 238-240 (1995).
