# Supplementary material for: Influence of atomic site-specific strain on catalytic activity of supported nanoparticles
Source: Nat Commun. 2018 Jul 13;9:2722. doi: 10.1038/s41467-018-05055-1 (PMC6045581; doi:10.1038/s41467-018-05055-1)
Supplement: Supplementary file 2 — Descriptions of Additional Supplementary Files [file 41467_2018_5055_MOESM2_ESM.pdf]

Descriptions of Additional Supplementary Files

File Name: Supplementary Movie 1
Description: Raw series of 148 HAADF STEM images of a Pt NP supported on alumina. Playback is eight times faster than the acquisition.

File Name: Supplementary Movie 2
Description: Series of 148 HAADF STEM images of a Pt NP supported on alumina after non-rigid image registration. Playback is eight times faster than the acquisition.
